# Rapid Mitochondrial Genome Evolution through Invasion of Mobile Elements in Two Closely Related Species of Arbuscular Mycorrhizal Fungi

**DOI:** 10.1371/journal.pone.0060768

**Published:** 2013-04-18

**Authors:** Denis Beaudet, Maryam Nadimi, Bachir Iffis, Mohamed Hijri

**Affiliations:** Département de Sciences Biologiques, Institut de Recherche en Biologie Végétale, Université de Montréal, Montréal, Québec, Canada; Institut de Genetique et Microbiologie, France

## Abstract

Arbuscular mycorrhizal fungi (AMF) are common and important plant symbionts. They have coenocytic hyphae and form multinucleated spores. The nuclear genome of AMF is polymorphic and its organization is not well understood, which makes the development of reliable molecular markers challenging. In stark contrast, their mitochondrial genome (mtDNA) is homogeneous. To assess the intra- and inter-specific mitochondrial variability in closely related *Glomus species*, we performed 454 sequencing on total genomic DNA of *Glomus sp.* isolate DAOM-229456 and we compared its mtDNA with two *G. irregulare* isolates. We found that the mtDNA of *Glomus sp.* is homogeneous, identical in gene order and, with respect to the sequences of coding regions, almost identical t*o G. irregulare*. However, certain genomic regions vary substantially, due to insertions/deletions of elements such as introns, mitochondrial plasmid-like DNA polymerase genes and mobile open reading frames. We found no evidence of mitochondrial or cytoplasmic plasmids in *Glomus* species, and mobile ORFs in *Glomus* are responsible for the formation of four gene hybrids in *atp6*, *atp9*, *cox2*, and *nad*3, which are most probably the result of horizontal gene transfer and are expressed at the mRNA level. We found evidence for substantial sequence variation in defined regions of mtDNA, even among closely related isolates with otherwise identical coding gene sequences. This variation makes it possible to design reliable intra- and inter-specific markers.

## Introduction

Arbuscular mycorrhizal fungi (AMF) are plant root-inhabiting obligate symbionts that form symbiotic associations with approximately 80% of plant species [1], [2]. This symbiosis helps plants to acquire nutrients and protects them from soil-borne pathogens [3], [4] by inducing plant resistance [5]–[8] or inhibiting pathogen growth [9]. In return, plants provide carbohydrates, which AMF cannot acquire from extracellular sources. They are an important component of soil microbial communities, as they are able to exchange their genetic material between compatible isolates through a process called anastomosis [10]. The latter have been hypothesized to be an important factor in maintaining the genetic diversity found in Glomeromycota and to attenuate the effect of genetic drift within a population [10]–[14]. AMF are currently thought to reproduce clonally, based on the absence of a recognizable sexual stage (or apparatus). However, this hypothesis has been challenged by the identification of many orthologues of sexually-related genes [15]–[17], which suggests at least the presence of cryptic recombination. AMF spores and hyphae are multinucleated, but their true genetic organization is currently under debate [11], [18]–[21]. However, evidence strongly suggests that nuclei can be genetically divergent within an AMF individual. Thus, AMF are characterized by considerable within-isolate nuclear genetic diversity even at the expression level [22]. The presence of such diversity in AMF individuals/populations [22], [23], combined with a lack of molecular data, have hindered the use of nuclear markers to assess questions on community structure, diversity and function. In contrast, AMF mitochondrial (mt) DNA is homogeneous within single isolates [24], [25], making it a good target for marker development. Following this logic, the mitochondrial large subunit (LSU) rRNA gene has been explored for its usefulness as a marker [24], [26], [27], although determining its specificity at the isolate level is still challenging for all AMF taxa aside from the model species *G. irregulare*.

Comparative AMF mitochondrial genomics has been proposed as an approach to open up new possibilities for development of strain-specific molecular markers [25], [28], [29] given that the type of mitochondrial marker necessary to establish specificity at different divergence levels may vary. This approach has been shown to be a powerful tool for the study of evolutionary relationships among lower fungi [30]. Unfortunately, only three AMF mitochondrial genomes had been published until recently, including that of *Glomus intraradices* [25], [29] (renamed to *G. irregulare* [31] and changed again recently to *Rhizophagus irregularis* based on an exhaustive molecular phylogeny of rRNA genes [32]; in the present paper, we will use the older nomenclature) as well as those of two distant AMF species, *Gigaspora rosea* and *Gigaspora margarita* [33], [34]. Compared to *G. irregulare*, the Gigasporaceae genomes have an inflated mitochondrial genome size that is mainly the result of extended intergenic regions. These regions are not syntenic and both genomes harbor *cox1* and *rns* genes with exons encoded on different strands, whose products are joined at the RNA level through either trans-splicing events of group I introns, or base-pairing. The mitochondrial protein sequences in the dataset were sufficient to confirm the phylogenetic relationship of AMF with *Mortierellales* as a sister group. This shows that a broader sampling of AMF mtDNA can answer questions about the evolution of these ecologically important fungi. Formey et al. (2012) have recently sequenced four isolates of *G. irregulare* [29] and were able to develop isolate-specific markers using variable regions That were created by the insertion of mobile elements.

Those elements, including linear or circular plasmids and mobile ORF encoding endonucleases (mORFs), are present in a broad range of fungal mitochondrial genomes (For review see [35]). Plasmids are autonomously-replicating circular or linear extrachromosomal DNA molecules. They are found in three broad types: circular plasmids encoding a DNA polymerase gene (*dpo)* [36], linear plasmids with terminal inverted repeats encoding either a *dpo* or *rpo* (RNA polymerase) gene or both [37], and retroplasmids, which usually encode a reverse transcriptase [38]. Free linear or circular plasmids encoding *dpo* can be present in the mitochondria of fungi [36] and plants [39]. Segments have been shown to integrate within the mtDNA of fungi [40]–[42], but plasmid-related *dpo* insertions tend to fragment, shorten (since they are not selected for) and eventually disappear from mitochondrial genomes. Plasmid-related *dpo* insertions have been reported in the AMF *Gigaspora rosea*, but are virtually absent from the closely related paraphyletic zygomycetes. The mobility of mORFs, elements that thrive in *Glomus*, is mediated by the site-specific DNA endonuclease they encode. This endonuclease cleaves ORF-less alleles by creating a double-strand break in DNA and initiates the insertion and fusion of the mobile element. The same process, called *intron homing*, has been proposed for group I introns [43]. Several lines of phylogenetic evidence support the hypothesis of the evolutionary-independent ancestral origins of mORFs [44]. These highly mobile elements have the ability to carry group I introns [45], intergenic sequences [46], and coding sequences [47]. The first reported case of mitochondrial gene transfer caused by those elements was a mORF-mediated insertion of a foreign *atp6* carboxy-terminal in the blastocladiomycete *Allomyces macrogynus* [48].

The present study compared the mitochondrial genomes of the newly sequenced AMF species *Glomus sp.* DAOM226456 (a *Glomus diaphanum* like species based on spore morphology) with two isolates of the closely related *G. irregulare*. Along with a highly divergent intron insertion pattern, we found insertions of plasmid-related DNA polymerase and propagation of mobile open reading frame (mORFs) encoding endonucleases in *Glomus* mtDNAs. Our findings have brought to light the first evidence of AMF interspecific exchange of mitochondrial coding sequences entailing formation of gene hybrids in *Glomus sp. atp6*, *atp9* (coding for the subunit 6 and 9 of the ATP synthetase complex), *cox2* (cytochrome C oxidase subunit 2) and *nad3* (NADH dehydrogenase subunit 3) genes.

## Materials and Methods

### Fungal material

Spores and mycelium of *Glomus sp.* (DAOM-229456) and *G. irregulare* (DAOM 197198) were cultivated *in vitro* on a minimal (M) medium with carrot roots transformed with *Agrobacterium rhizogenes*, as described in the literature [49]. The medium was liquefied using a 0.82 mM sodium citrate and 0.18 mM citric acid extraction buffer solution. The resulting fungal material was further purified by hand under a binocular microscope, to remove root fragments.

### DNA extraction

Spores and mycelium were suspended in 400 µL of the DNeasy Plant Mini Kit AP1 buffer (Qiagen) and crushed with a pestle in 1.5 ml microtubes, and the DNA was purified according to the manufacturer's recommendations. Purified DNA in a final elution volume of 40 µL was stored at −20°C until use.

### RNA extraction

Fresh *Glomus sp.* fungal material was harvested from *in vitro* cultures. RNA extraction was performed using an E.Z.N.A. Fungal RNA Kit (Omega Biotek) according to manufacturer's recommendations. Total RNA was treated with Turbo DNase (Applied Biosystems) for 30 min at 37°C to remove residual DNA fragments that could interfere with downstream applications. In order to prevent chemical scission of the RNA during heat inactivation of the DNase at 75°C for 15 min, EDTA was added at a final concentration of 15 mM. In total, 40 µl of 100 ng/µl RNA was collected and stored at −80°C until use. The RNA concentration was determined using a Nanophotometer Pearl (Implen).

### cDNA synthesis

From the total RNA previously extracted, 500 ng were used for cDNA synthesis with the SuperScript III reverse transcriptase kit (Life Technologies, Canada) according to manufacturer's recommendations, using oligo dT. The only change from these recommendations was the addition of MgCl_2_ to a final concentration of 15 mM to compensate for the EDTA added in the previous step. In order to remove RNA complementary to the cDNA, 1 µl of RNase ONE ribonuclease (Promega, Canada) was added to the cDNA and incubated at 37°C for 20 min. The resulting cDNA was stored at −20°C until use.

### Polymerase chain reaction (PCR)

The proposed intergenic markers to discriminate between *G. irregulare* DAOM197198 and *Glomus sp.* were tested by PCR using the KAPA2G Robust Hotstart ReadyMix PCR kit (KapaBiosystems, Canada). The specific primers used were respectively *rnl-cox2*_197198_spec_F (5′-AAAGGAATTACATCGATTTA-3′), *rnl-cox2*_197198_spec_R (5′- ACAAGAAGGTTTGCATCGCTA-3′), *nad6-cox3*_dia_spec_F (5′- CCACTAGTTAAGCTACCCTCTA-3′) and *nad6-cox3*_dia_spec_R (5′- AATCATACCGTGTGAAAGCAAG -3′). The variable length primers were *rnl-cox2*_197198_size_F (5′- TAGGGATCAGTACTTTAGCCAT -3′), *rnl-cox2*_197198_size_R (5′- TCCTTACGGTATGAATGGTAAG -3′), *rnl-cox2*_dia_size_F (5′- AGACTTCTTCAGTTCCACAATCA -3′) and *rnl-cox2*_dia_size_R (5′- ATGGCTAAAGTACTGATCCCTAC -3′). For 40 µl of PCR reaction volume, 12 µl of water, 20 µl of 2× PCR buffer, 3.5 µl of (5 µM) forward and reverse primers, and 1 µl of DNA were added. Cycling parameters were 94°C/3 min, followed by 38 cycles of: 94°C/30 sec, 54°C/25 sec, 72°C/45 sec and a final elongation at 72°C. PCR products were separated by electrophoresis in a 1.5% (w/v) agarose gel and visualized with GelRed under UV light.

### Reverse transcriptase – polymerase chain reaction (RT-PCR)

Our objective with regard to PCR reactions on cDNA was to assess which regions of the gene hybrid reported in *atp6*, *atp9*, *cox2* and *nad3* were expressed at the mRNA level. For each of the four hybrids, a forward primer designed in the conserved ‘core’ structure of the gene (*atp6*_core_F: 5′-AGAGCAGTTTGAGATTGTTAAG-3′, *atp9*_core_F: 5′-CTGGAGTAGGAGTAGGGATAGT-3′, *cox2*_core_F: 5′-CATGGCAATTAGGATTTCAAGA-3′ and *nad3*_core_F: 5′-TCGTTCCTTTGTTCGTGCTA-3′) was used in combination with three reverse primers designed respectively in the inserted C-terminal (*atp6*_insert_R1: 5′-AGCCTGAATAAGTGCAACAC-3′, *atp9*_insert_R1: 5′- GTAAGAAAGCCATCATGAGACA-3′, *cox2*_insert_R1: 5′-TGAGAAGAAAGCCATAACAAGT-3′ and *nad3*_insert_R1: 5′-AGAAGTATGAAAACCATAGCAATC-3′), the mobile ORF (*atp6*_mORF_R2: 5′-AGTCTTCGAATATACTGGCAG-3′, *atp9*_mORF_R2: 5′-TGTCGAGTCTCCAAAGTATGT-3′, *cox2*_mORF_R2: 5′-ACTGAATTCCTGTGTTTCGATCT-3′ and *nad3*_mORF_R2: 5′-TGACGAATGGTTAGACGATGT-3′) and the native C*-terminal portion of the corresponding gene (*atp6*_native_R3: 5′-CGTACCGTCGTAACAAGTAGA-3′, *atp9*_native_R3: 5′-CCATCATTAAGGCGAATAGA-3′, *cox2*_native_R3: 5′-CTAACAAACTCCCGACTATTACCT-3′ and *nad3*_native_R3: 5′-AGAATGAAGACCATTGCAAC-3′). To verify that there was no residual mitochondrial DNA in the cDNA, the primers Ctrl_positive_*nad5*exon4_689F (5′-ACCATTCTGTTATGTTCTAATGT-3′) and Ctrl_positive_*nad5*exon4_689R (5′-GTCTGACTTAGCAGGTTAGTTAAG-3′) were designed in *nad5* exon 4 and used as a positive control on cDNA and negative control on RNA. The RT-PCR reactions were carried out using the KAPA2G Robust Hotstart ReadyMix PCR kit (KapaBiosystems, Canada) as described above in the PCR section.

### Cloning

Cloning reactions were performed on each successful RT-PCR amplification. The ligation reactions were done using the pGEM-T Easy Vector Systems kit (Promega, Canada) according to manufacturer's recommendations. The transformation was carried out in *E. coli* DH5 alpha competent cells. Bacterial colonies were screened via PCR using T7 and SP6 universal primers as described in the PCR section.

### Sequencing, assembly and gene annotation

*Glomus* sp. total DNA was sequenced using 454 Titanium Flex shotgun technology (one plate) and the respective resulting 1,078,190 reads were assembled with Newbler (Genome Quebec Innovation Center, McGill University, Montreal, Canada). Gene annotation was performed with MFannot (http://megasun.bch.umontreal.ca/cgi-bin/mfannot/mfannotInterface.pl), followed by manual inspection and introduction of missing gene features as described in Nadimi et al., (2012). *G. irregulare* isolates 494 and DAOM-197198 mtDNAs (accession numbers FJ648425 and HQ189519 respectively) were used for comparison. Sequencing of the cloned RT-PCR products was performed on the same sequencing platform, using Sanger technology with T7 and SP6 universal primers.

### Phylogenetic analysis

For each gene of interest (*atp6, atp9, cox2* and *nad3*) in 12 AMF species, the dataset contains the corresponding C*-terminal for: *Glomus sp.* DAOM-229456, *G. irregulare* isolate 494, *G. irregulare* DAOM-197198, *G. irregulare* DAOM-240415, *G. irregulare* DAOM-234179, *G. irregulare* DAOM-234328, *G. irregulare* DAOM-213198, *Glomus sp.* DAOM-240422, *G. fasciculatum* DAOM-240159, *G. aggregatum* DAOM-240163, *G. cerebriforme* DAOM-227022, *Gigaspora rosea* DAOM-194757 (accession number JQ693396) and 3 selected fungal representatives: *Mortierella verticillata* (accession number AY863211), *Smittium culisetae* (accession number AY863213) and *Rhizopus oryzae* (accession number AY863212). The sequences were deposited in databases under the accession numbers: JX074786-JX074817. The reference phylogeny was constructed using the concatenated ‘core’ sequence (without the C*-terminal portion used previously) of the same four genes. The DNA sequence alignments and the inference of maximum likelihood trees using GTR+G (with five distinct gamma categories) were performed using the integrated program MEGA version 5 [50]. Bootstrap resampling (1000 replicates) was carried out to quantify the relative support for each branch of the trees. Bayesian analysis were done using MrBayes version 3.2 using the GTR+G model (with five distinct gamma categories), four independant chains, one million cycles, tree sampling every 100 generations and a burn-in value of 25%.

## Results and Discussion

### *Glomus sp.* genome organization and structure

**Figure 1:**
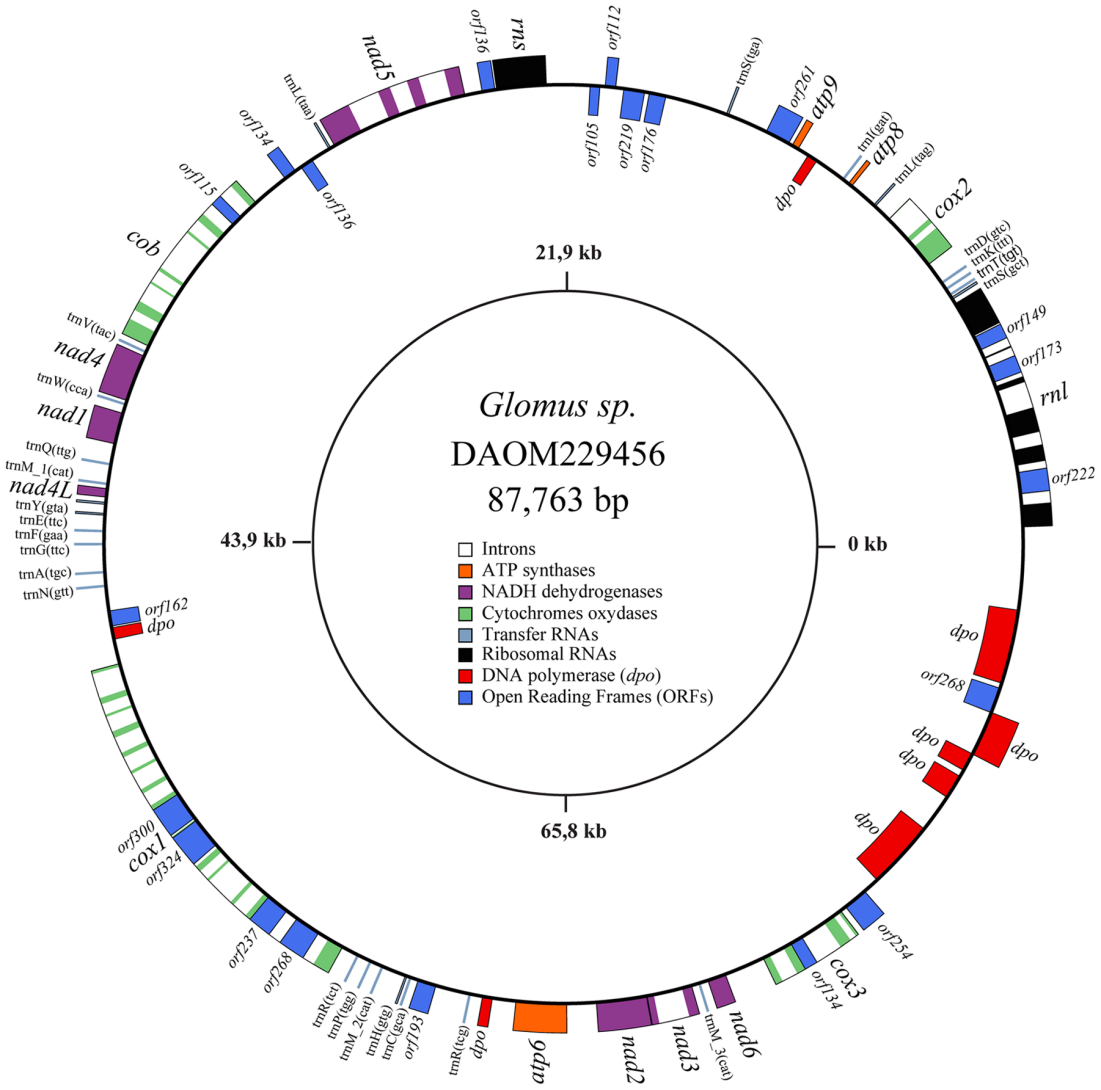
The *Glomus sp.* 229456 mitochondrial genome circular-map was opened upstream of *rnl*. Genes on the outer and inner circumference are transcribed in a clockwise and counterclockwise direction, respectively. Gene and corresponding product names are *atp6, 8, 9,* ATP synthase subunit 6; *cob*, apocytochrome b; *cox1–3*, cytochrome c oxidase subunits; *nad1–4, 4L, 5–6*, NADH dehydrogenase subunits; *rnl, rns*, large and small subunit rRNAs; A–W, tRNAs, the letter corresponding to the amino acid specified by the particular tRNA followed by their anticodon. Open reading frames smaller than 100 amino acids are not shown.

The complete sequence of the *Glomus sp.* 229456 mt genome was a double-stranded circular DNA molecule, exempt of polymorphism, with a size of 87,763 bp. The annotated sequence of *Glomus sp.* was deposited in GenBank under the accession number JX065416. Its mtDNA harbors the typical set of 41 mitochondrial genes found in other AMF (two rRNAs, 14 protein coding genes (PCGs) and 25 tRNAs). The PCGs include three ATP synthetase (*atp*), one cytochrome b (*cob*), three cytochrome C oxydase (*cox*) and seven NADH dehydrogenase (*nad*) genes. Also, 19 ORFs and 31 introns are inserted in this newly sequenced mt genome (Figure 1).

### Comparative view of three *Glomus* mtDNAs

**Figure 2:**
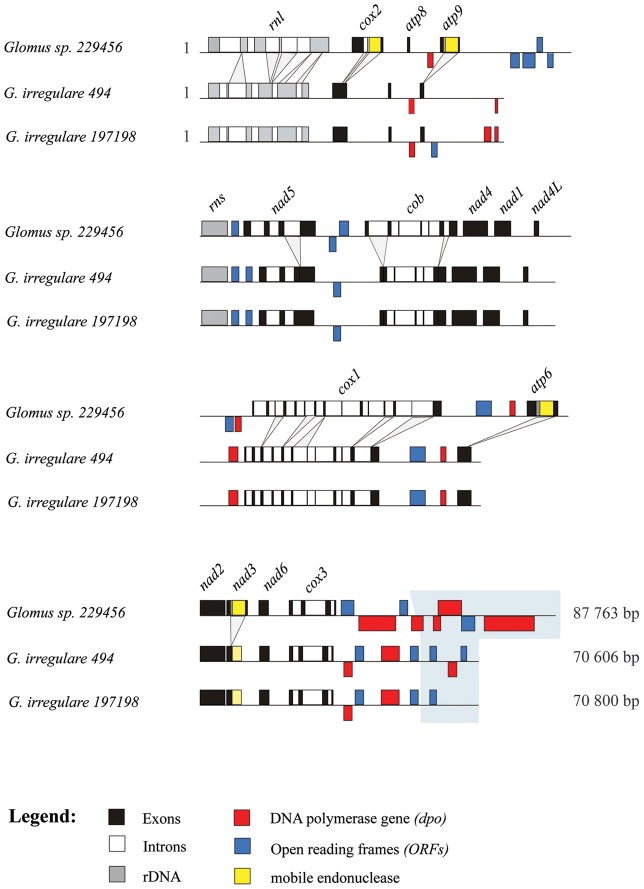
Comparative view of the three mitochondrial genomes linear map where the exons (black), introns (white), rDNA (gray), *dpo* plasmid insertions (red), ORFs (blue) and mobile endonuclease (yellow) are represented. Divergence in intron insertion pattern is indicated by projections. A hyper-variable region in the *cox3-rnl* intergene is boxed in grayscale.

The gene content in *Glomus sp.* and *G. irregulare* mitochondrial genomes is similar to that found in zygomycetes, except for *rps3* and *rnpB.* The mtDNAs of both AMF species have the same gene order, and all genes are transcribed from one strand with very similar coding regions except for the insertion of mobile ORF elements (mORFs) in the *atp6, atp9*, *cox2* and *nad3* genes of *Glomus sp.* (Figure 2). However, there are many differences in the number of introns, some of which carry more substantial sequence differences than do the coding sequences. The differences in the presence of introns and mORFs explain the inflated genome size of 87,763 bp in *Glomus sp.*, as compared to 70,800 bp in *G. irregulare* 197198 (Table 1). *Glomus sp*. *cox1* intron 8 is the homolog of an intron inserted at the same position in *Rhizopus oryzae* and angiosperms (with 76 and 79% of sequence identity, respectively) [28]. *G. irregulare cox1* intron 7 is also inserted at the same position, but has an eroded ORF encoding the homing endonuclease gene, and thus also shares identity with the intron RNA secondary structure of *R. oryzae* and plants. The plant *cox1* intron was thought to have been acquired from a fungal donor, due to the proximity of its clade to that of fungi rather than to the non-vascular plant *Marchantia*. Knowing the extent to which the intron has spread in angiosperms [51], [52], it would be interesting to see whether such an invasion has also occurred within the Glomeromycota phylum.

**Table 1:** Gene and intron content in AMF and selected fungal mtDNAs.

	Genes										
Species	*rnl, rns*	*atp 6, 8, 9*	*cob*	*cox 1, 2, 3*	*nad 1*–*6* a	*trn A*–*W*	*rnpB*	*rps3*	*ORFs* b	*Intron I* c	*Intron II* c
*Glomus Sp. 229456*	2	3	1	3	7	25	0	0	19	31	1
*Glomus irregulare 494*	2	3	1	3	7	25	0	0	8	26	0
*Glomus irregulare 197198*	2	3	1	3	7	25	0	0	8	26	0
*Gigaspora rosea*	2	3	1	3	7	25	0	0	4	13	1
*Smittium culisetae*	2	3	1	3	7	26	1	1	3	14	0
*Mortierella verticillata*	2	3	1	3	7	28	1	1	7	4	0
*Rhizopus oryzae*	2	3	1	3	7	23	1	0	4	9	0
*Allomyces macrogynus*	2	3	1	3	7	25	0	1	4	26	2
*Saccharomyces cerevisiae* d	2	3	1	3	0	25	1	1	3	9	4

a Includes *nad1*, *nad2*, *nad3*, *nad4*, *nad4L*, *nad5* and *nad6*.

b Only ORFs greater than 100 amino acids in length are listed, not including intronic ORFs and *dpo* and *rpo* fragments.

c Intron I and Intron II denote introns of group I and group II, respectively.

d *S. cerevisiae* strain FY 1679 [57].

**Figure 3:**
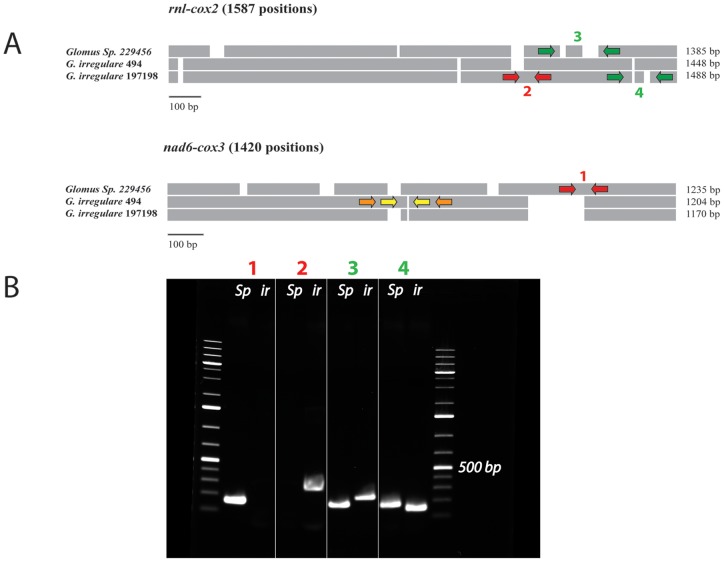
A) Schematic alignment representation of two mitochondrial intergenic regions (*rnl-cox2* and *cox3-nad6*) showing the presence of numerous insertions and deletions (indels). The red arrows indicate the approximate position of the PCR primers that yield strain-specific markers, while the green arrows indicate the position of PCR primers that produce a size-specific marker. The yellow and orange arrows indicate potential regions to design, respectively, specific and size-specific markers in *G. irregulare* 494. **B**) Agarose gel electrophoresis figure showing the PCR results of the proposed markers on *Glomus sp.* 229456 (*Gs*) and *G. irregulare* DAOM197198 (*Gi*) respectively for each marker. Marker 1 shows the *Glomus sp.* specific amplification (156 bp), while marker 2 shows the *G. irregulare* 197198 specific marker (263 bp). The size-specific marker 3 yield a length of 160 bp for *Glomus sp.* and 226 bp for *G. irregulare* 197198. Finally, the size-specific marker 4 yield a length of 159 bp for *Glomus sp.* and 131 bp for *G. irregulare* 197198.

Further, intergenic regions differ substantially in sequence: some are identical while others show signs of very fast, substantial changes including point mutations, insertions, deletions and inversions (Figures 2 and 3). Most of these differences occur in the *cox3-rnl* intergene, a large hyper-variable region that has been invaded by *dpo* fragments. The variations observed in intergenic regions provide an opportunity to develop species- specific molecular markers as shown in Figure 3, and even isolate-specific markers or methods allowing reliable identification and/or quantification of these fungi. Lack of efficient and powerful molecular markers for AMF identification and quantification constitutes a major problem that limits the analysis of population genetics and field studies in AMF. Mitochondrial DNA is homogeneous within the AMF individuals studied to date, but evidence of genetic polymorphism between *G. irregulare* isolates has been observed in intergenic regions. They harbor highly conserved genes as well as highly variable regions, which promises to facilitate AMF barcoding at different taxonomic levels, an analysis that is currently challenging to carry out using nuclear genes. Hyper-variable intergenic regions with eroded *dpo* insertions and indels in intergenic regions constitute useful mitochondrial areas on which to focus attention in order to develop suitable markers for discriminating isolates of the same species. Intron insertion pattern variations, genome reorganizations (such as gene shuffling) and coding region divergences will make it possible to distinguish between different AMF species, genera and families.

Our 454 pyrosequencing data and direct PCR sequencing showed that *G. irregulare* DAOM197198 and *Glomus sp.* mtDNAs are homogeneous, meaning that all the mitochondrial genomes in a given isolate are essentially identical, in stark contrast to the nuclear genomes. Our results confirm the previous report by Lee *et al.* (2009) suggesting homoplasmy in the first completed Glomeromycota mitochondrial genome of the AMF *G. intraradices* (*G. irregulare* isolate 494). A rapid and effective mitochondrial segregation mechanism was suggested to explain those findings. It was previously demonstrated that isolates of the same species can exchange nuclear material through anastomosis [10], but exchange of divergent mitochondrial haplotypes has yet to be shown. This leads us to question whether polymorphism does indeed occur through anastomosis, and for how many generations mitochondrial heteroplasmy is maintained.

### Rapid expansion of plasmid-like DNA polymerase sequences in *Glomus*

Plasmid-related DNA polymerase genes are found in mobile mitochondrial plasmids that occur either as free linear or circular DNAs, and have been shown to also insert into mtDNA (for review see [35]). One striking feature in the comparison of the two closely related *Glomus* species is the presence of numerous *dpo* insertions in the intergenic regions of their mtDNA (Figure 2). All three *Glomus* mtDNAs contain a large number of *dpo* fragments, most of which are substantially divergent in sequence and therefore are most likely the result of independent plasmid insertion events. Even the two *G. irregulare* (isolates 494 and DAOM197198), otherwise almost identical in sequence, differ in *dpo* sequence, which supports the interpretation that *dpo* insertion occurs repeatedly and frequently through evolutionary time. A *bona fide* and complete *dpo* gene is present in *Glomus sp*., and its sequence is different from those in *G. irregulare* isolates. Because of its complete length, it most likely results from a recent insertion event. There is no evidence that *dpo* is functional when inserted in mtDNA. As in numerous other cases, *dpo* coding regions are fragmented in *Glomus* and occur on both strands, representing a good indicator of a genomic region experiencing little if any selective evolutionary constraints.

The source of the *dpo* insertions in *Glomus* mtDNA remains elusive. We did not find any free mitochondrial plasmids in our *Glomus sp.* and *G. irregulare* isolate DAOM197198 shotgun data (combining nuclear and mitochondrial DNAs), as we did for *Gigaspora rosea*, where a 3582 bp contig with high sequence coverage was found [34]. However, since the *Glomus* strains used in this study come from aseptic *in vitro* cultures, and even though the *G. rosea* fungal material was extracted from *in vivo* greenhouse pot cultures, we cannot rule out the possibility that an environmental vector is the source of *dpo* plasmids and is responsible for their propagation in *G. rosea*. Interestingly, *dpo* plasmids have been found to occur in numerous plants, notably in *Daucus carota* [53] which is used as a host plant for AMF cultures *in vitro*. The obligate biotrophic dependence of AMF on plants could be one of the reasons that *dpo* insertions are most abundant in *Glomus* mtDNAs yet virtually absent in mitochondrial sequences of the Blastocladiomycota (except for a single 100 amino acid long fragment occurrence in *Smittium culisetae* mtDNA), which is the closest phylogenetic group to the Glomeromycota.

Mobile element insertions have been shown to trigger genomic rearrangements such as gene shuffling through homologous recombination [54] and even genome linearization [35], [55], [56]. Whenever sequence repeats occur, more than one genome conformation may exist, but we have no evidence that this happens in *Glomus* mtDNA. It would be interesting to examine whether numerous recent *dpo* insertions with high sequence similarity might act as genomic repetitions and give rise to genome reorganization in closely related AMF species. Integrated plasmid segments within mitochondrial genomes, even though they are neutral or cryptic, could promote genomic rearrangements.

### Mobile ORF elements (mORFs) in *Glomus*

Although most ORF-encoding endonuclease genes are inserted in introns where they have been shown to play a role in propagation, they can also be present in genes in which their evolutionary impact is less obvious. We identified numerous mORFs encoding endonuclease genes unique to *Glomus sp.* isolate DAOM-229456 mtDNA. When we annotated the sequences of the *atp6*, *atp9*, *cox2* and *nad3* genes, we observed that they all have a peculiar organization. Indeed, these genes harbor a carboxy-terminal duplication (C*-terminal) that was found downstream of a mORF insertion. For example, in the *atp6* gene, the duplicated portion of the C-terminal was found about 1000 bp downstream, following an inserted LAGLIDADG endonuclease ORF. When we compared the DNA sequence of the C*-terminal portion with the corresponding sequence of *G. irregulare* isolate 494, a close relative to *Glomus sp.*, we found a 91.2% nucleotide identity. In contrast, the comparison between the *Glomus sp. atp6* duplicated carboxy-terminals (C-terminal and C*-terminal) showed a low sequence identity of 63.5%. Interestingly, comparison of the *Glomus sp*. C*-terminal amino acid sequences with the corresponding portion in *G. irregulare* showed 100% identity, indicating that the mutations observed in DNA are all synonymous. However, the comparison of the amino acid sequences of *Glomus sp*. *atp6* carboxy-terminals showed 91% identity.

**Figure 4:**
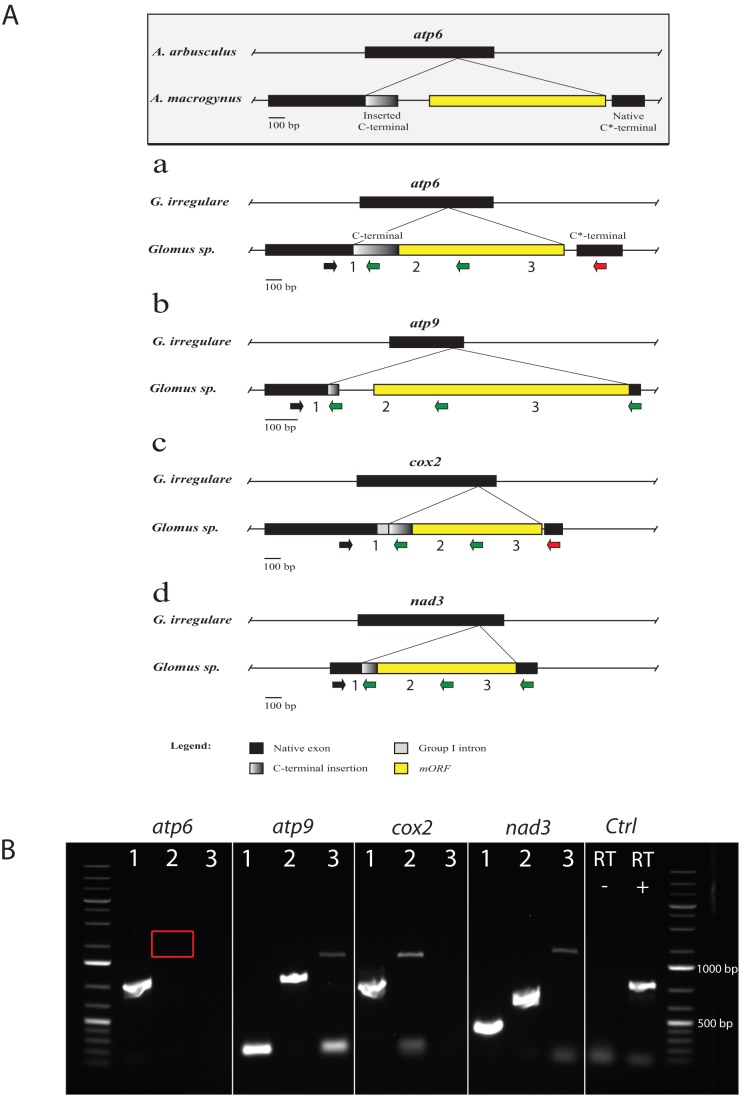
Comparison of gene hybrids atp6, atp9, cox2 and nad3. **A**) The *atp6* gene hybrid reported for *Allomyces macrogynus* (grayscale, boxed) is used as a reference in a comparison of the most similar *atp6* (a), *atp9* (b), *cox2* (c) and *nad3* (d) genes in *Glomus sp.* mtDNA. Each occurrence is put in perspective with the gene of a close relative (either *Allomyces arbusculus* or *G. irregulare*) in order to show the insertion point of the foreign element with the projections. Exons are in black, while the inserted foreign C-terminal is shaded in gray. The mobile endonuclease element is in yellow. For each gene, the black arrow indicates the position of the forward primer used in the downstream RT-PCR experiment in combination with three different reverse primers. The green arrows indicate expression at the RNA level of the corresponding portion of the gene, while the red arrows indicate a negative amplification. **B**) Agarose gel electrophoresis figure showing the RT-PCR results. For each gene hybrid, the expression at the RNA level was tested using a forward primer in the conserved gene core and a reverse primer respectively in the inserted C-terminal (1), the mobile endonuclease (2) and the native C*-terminal portion (3). Primers in *nad5* exon 4 were used as a positive control on cDNA (RT +) and negative control on RNA (RT −). The expected size of the amplified fragments was: *atp6* inserted C-terminal (684 bp), *atp9* inserted C-terminal (149 bp), *atp9* mORF (717 bp), *atp9* native C*-terminal (1085 bp), *cox2* inserted C-terminal (938 bp), *cox2* mORF (1291 bp), *nad3* inserted C-terminal (261 bp), *nad3* mORF (597 bp), *nad3* native C*-terminal (1183 bp) and the positive control in *nad5* exon 4 had an expected amplicon size of 689 bp. The red box indicates a faint band that is present on the gel.

Surprisingly, when we designed a forward primer in the upstream sequence (5′ gene portion) and two reverse primers in the C-terminal and C*-terminal respectively, we found that the C-terminal is transcribed with the upstream sequence resulting in a putative hybrid transcript while the C*-terminal was not expressed into mRNA. Thus we hypothesized that the C-terminal portion could have been acquired from a donor through horizontal gene transfer (HGT). We also observed similar organization in *atp9*, *cox2* and *nad3* genes of *Glomus sp.* where the carboxy-terminal portion (C*-terminal) was replaced partially or completely by one carried by a mORF (C-terminal) encoding a LAGLIDADG endonuclease (except in *atp9*, a GIY-YIG family endonuclease) (Figure 4A: a, b, c and d). In *atp6*, the insert lacks a stop codon and the ORF is in phase with the native gene. In *atp9*, the insert, along with the mORF, completely replaces the native 3′ end, while in *cox2* and *nad3* only a portion of the carboxy-terminal is replaced (Table 2). The resulting gene hybrids are expressed at the mRNA level in all four cases as shown in Figure 4B. After sequencing of the cDNA bands, we found that the mORF and the inserted C-terminal are integral parts of the transcript in all four genes. However, in *atp6* and *cox2*, the native C*-terminal was not expressed into mRNA.

**Table 2:** Description of the gene hybrids found in *Glomus sp.* 229456 mtDNA.

	*atp6*	*atp9*	*cox2*		*nad3*
Total length	1569	1171	1894		1242
CDS length	1569	225	837		1242
Features					
Group I intron	-	-		[684–922]	
Inserted C-terminal	[537–774] 1	[175–225] 1		[923–1018] 1	[194–313] 1
*mORF*	[550–1567] 1	[334–1119] 1		[1187–1738] 1	[451–867] 1
Native C*-terminal	[1582–1860]	[1120–1171] 1		[1739–1894]	[1116–1238] 1
Remarks	C-terminal and m*ORF* in phase with native gene	C-terminal in phase with native gene.		Partial inserted C-terminal in phase with native gene.	C-terminal and m*ORF* in phase with native gene.

1 Gene hybrid features that are expressed at the mRNA level (see Figure 4B).

These gene hybrid structures are similar to that of the *atp6* gene previously described in the *Allomyces macrogynus* (Figure 4, grayscale box), a species that belongs to the basal fungal phylum Blastocladiomycota [48]. The same scenario has also been observed in the *Rhizopus oryzae atp9* and *Mortierella verticillata cox2* genes [30]. These hybrids contain a carboxy-terminal duplication as well as a mORF encoding an endonuclease, which has been biochemically demonstrated to be responsible for the element mobility. In *Allomyces macrogynus*, the inserted C-terminal was shown to have been recently acquired by HGT based on the divergence in sequence it had with the native C*-terminal, while the latter had a perfect sequence identity with the corresponding gene portion of the closely related species *Allomyces arbusculus*.

The *Glomus sp. atp6, atp9*, *cox2* and *nad3* native C*-terminals showed higher nucleotide sequence identity to those of *G. irregulare* 494 (91, 98, 93 and 98%, respectively) than their duplicated C-terminal counterparts (64, 71 and 81 and 73%, respectively) (Figures S1, S2, S3, and S4 and Tables S1, S2, S3, and S4). However at the protein level, the comparison of the C*-terminal amino acid sequences of the *atp6*, *atp9, cox2* and *nad3* genes with the corresponding portion in *G. irregulare* 494 was 100% for *atp6, 94% for atp9, and* 100% for *cox2* and *nad3*. The high sequence identity of the native *Glomus sp.* C*-terminals with *G. irregulare 494,* is in stark contrast to the low similarity observed with the inserted C-terminal portions and points to a recent HGT event, as was described in *Allomyces spp.* [*48*]. However, the HGT hypothesis could likely apply to the *atp6* and *cox2* genes, since their native C*-terminal portion is no longer translated and could undergo rapid divergence. For the *atp9* and *nad3* hybrids, even though it is less parsimonious, the observed sequence divergence between the duplicated portions could have been caused by independent evolution following the mobile element insertion, since both are expressed in the mRNA transcript. It would also be interesting to see if some of the reported gene hybrids can still accomplish their functions at the protein level, given that the mORF and both C*-terminals are expressed in some cases. They are apparently expressed pseudogenes but post-translational modification mechanisms may be in place to ensure that the resulting protein is functional. We did not find a mORF-less copy of those genes that could have been transferred to the *Glomus sp.* nuclear genome that could explain a pseudogenization in *Glomus sp.* mtDNA.

**Figure 5:**
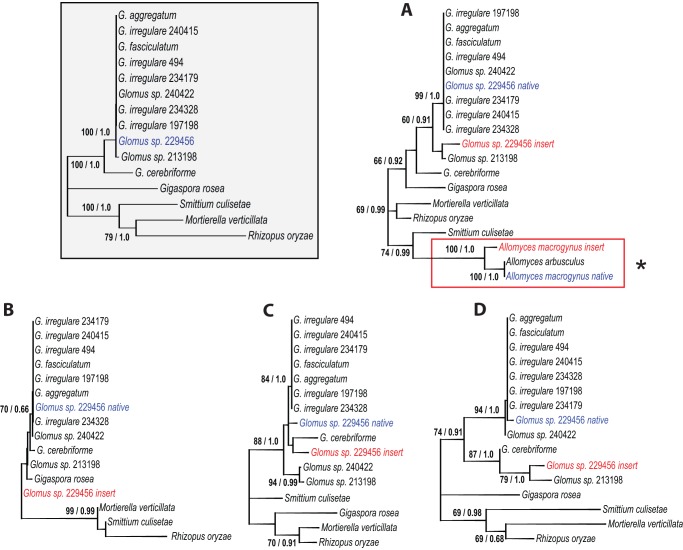
Unrooted maximum likelihood trees obtained with the GTR+G model (5 distinct gamma categories). The first number at branches indicates ML bootstrap values with 1000 bootstrap replicates and the second number indicates posterior probability values of a MrBayes analysis with four independant chains. Bayesian inference predict similar trees (not shown). The concatenated tree of the *atp6*, *atp9*, *cox2* and *nad3* ‘core’ genes (without the duplicated C*-terminal portion) (1489 alignment positions) of selected AMF representatives (grayscale boxed) are compared with those of the *atp6* (298 alignment positions), where the red box with the asterisk point out to the reference *Allomyces spp.* HGT event (Figure 4) (A), *atp9* (51 alignment positions) (B), *cox2* (106 alignment positions) (C) and *nad3* (120 alignment positions) (D) C*-terminals. The *Glomus sp.* native C*-terminals are in blue, while the inserted C-terminals are in red.

In regards to the HGT hypothesis, and in order to evaluate whether there is a plausible donor for the duplication, we compared the carboxy-terminal sequence of these genes with those in 11 *Glomus spp.* (to avoid redundancy we didn't add the *G. margarita* sequences since they are identical to *G. rosea*) and three phylogenetically related fungal representatives (Figure 5). In all four *Glomus sp.* gene hybrids (*atp6, atp9*, *cox2* and *nad3*), the native C*-terminal sequences cluster within the *Glomus spp.* group as expected given the reference phylogeny (Figure 5, grayscale box), thereby supporting a recent insertion of the foreign element. The *atp6* gene carboxy-terminal comparison (Figure 5A) shows that the mORF-derived C-terminal is related to a *Glomus sp.* isolate DAOM213198 with a moderate 60% bootstrap value. Surprisingly, in *atp9* (Figure 5B) the inserted C-terminal is even more distantly related to *Glomus spp.* than to *G. rosea*. In *cox2* (Figure 5C) the *Glomus sp.* inserted C-terminal and the more divergent AMF species *G. cerebriforme* are in the same cluster. Finally, the *nad3* C-terminal clustered with *Glomus sp.* 213198, as it was the case for *atp6,* with a 79% bootstrap value (Figure 5D). Also, the *nad3* gene shows high variability in length in *Glomus spp.,* due to the insertion of those elements.

In all four cases, the native *Glomus sp.* C*-terminal is nested within the *Glomus spp*. group and the inserted C-terminal is in a different cluster. Although it is difficult to pinpoint the donor of the sequence duplications, due to the possibly complex evolutionary history of those mobile elements with numerous insertion/loss events and 3′ end reshufflings, our data suggest HGT from a foreign AMF species, and thus the first reported occurrence in Glomeromycota. The presence of foreign DNA elements could potentially hamper mitochondrial gene phylogeny analysis unless the foreign C-terminals are carefully removed from the native portion of the gene.

## Conclusion

The inclusion of mitochondrial sequences from phylogenetically distant AMF species in the database is essential for developing a better understanding and classification of AMF within fungi. The mitochondrial genome comparison presented here for two closely related AMF species reveals substantial changes in mitochondrial gene sequences, resulting from *dpo* plasmid insertions and mobile ORFs invasions, along with intergenic sequence variation. This illustrates the importance of adding closely related species to the numerous isolates of the same species in the AMF mitochondrial genome collection. Comparative mitochondrial genomics, together with a broader sequencing effort in AMF, opens new avenues for the development of molecular markers at different evolutionary distances. It would be interesting to identify the source of plasmid-related DNA polymerase in AMF mtDNA, which should provide an estimate of the extent to which it is present within the Glomeromycota phylum and an assessment of the consequences on mitochondrial genome organization. Also, the mORF-carried foreign C-terminal described here represents the first reported evidence of HGT in AMF. The intimate relationship between AMF, the roots of their plant symbiont and soil microorganisms might be a perfect biological context to facilitate such transfers. To what extent the mobilome and HGT may have contributed to AMF evolution is a topic that merits exploration in future studies.

## Supporting Information

Figure S1 Multiple DNA sequence alignment of numerous AMF representatives of the atp6 native C-terminals along with the Glomus sp. 229456 putative foreign inserted C*-terminal.
(TIF)

Figure S2 Multiple DNA sequence alignment of numerous AMF representatives of the atp9 native C-terminals along with the Glomus sp. 229456 putative foreign inserted C*-terminal.
(TIF)

Figure S3 Multiple DNA sequence alignment of numerous AMF representatives of the cox2 native C-terminals along with the Glomus sp. 229456 putative foreign inserted C*-terminal.
(TIF)

Figure S4 Multiple DNA sequence alignment of numerous AMF representatives of the nad3 native C-terminals along with the Glomus sp. 229456 putative foreign inserted C*-terminal.
(TIF)

Table S1 Sequence identity matrix of the atp6 native C-terminals along with the Glomus sp. 229456 putative foreign inserted C*-terminal.
(DOC)

Table S2 Sequence identity matrix of the atp9 native C-terminals along with the Glomus sp. 229456 putative foreign inserted C*-terminal.
(DOC)

Table S3 Sequence identity matrix of the cox2 native C-terminals along with the Glomus sp. 229456 putative foreign inserted C*-terminal.
(DOC)

Table S4 Sequence identity matrix of the nad3 native C-terminals along with the Glomus sp. 229456 putative foreign inserted C*-terminal.
(DOC)
