# Supplementary figures and images for: Rapid Mitochondrial Genome Evolution through Invasion of Mobile Elements in Two Closely Related Species of Arbuscular Mycorrhizal Fungi
Source: PLoS One. 2013 Apr 18;8(4):e60768. doi: 10.1371/journal.pone.0060768 (PMC3630166; doi:10.1371/journal.pone.0060768)

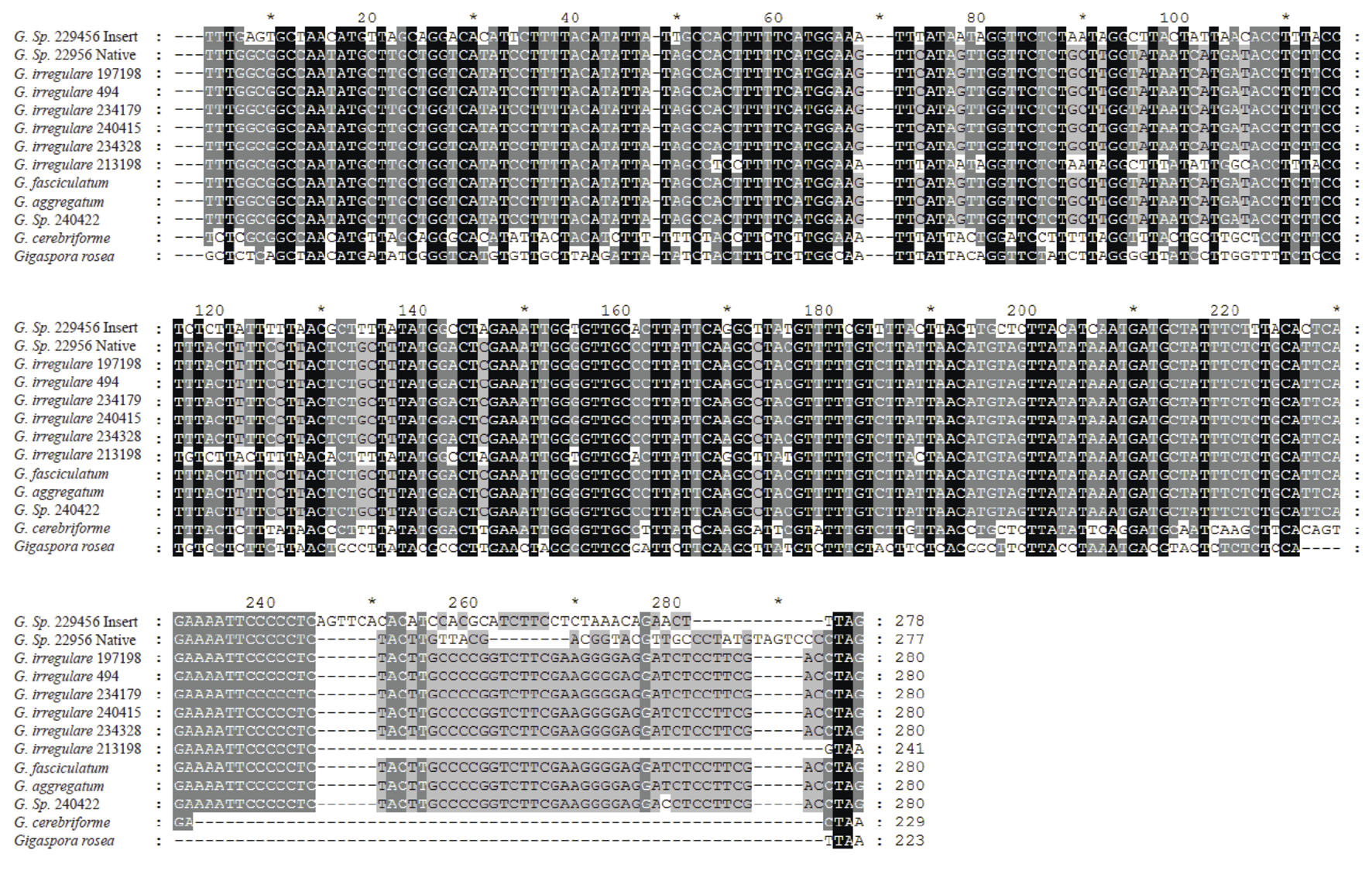

Supplement: Figure S1 — Multiple DNA sequence alignment of numerous AMF representatives of the atp6 native C-terminals along with the Glomus sp. 229456 putative foreign inserted C*-terminal. (TIF) [file pone.0060768.s001.tif]

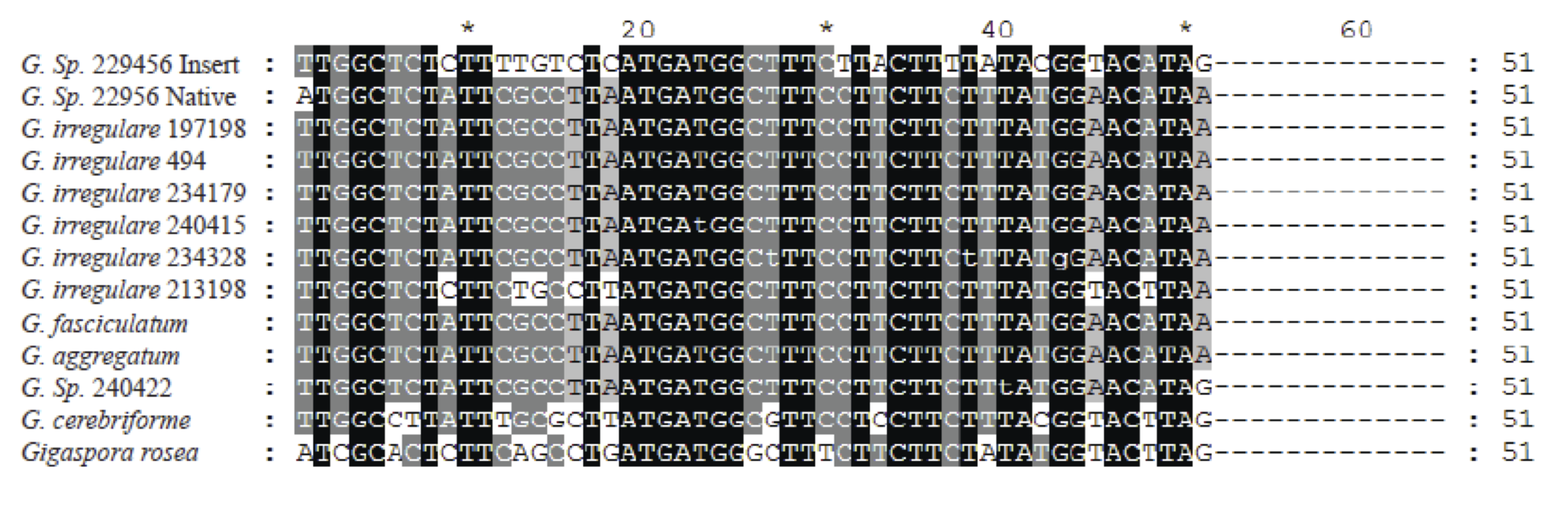

Supplement: Figure S2 — Multiple DNA sequence alignment of numerous AMF representatives of the atp9 native C-terminals along with the Glomus sp. 229456 putative foreign inserted C*-terminal. (TIF) [file pone.0060768.s002.tif]

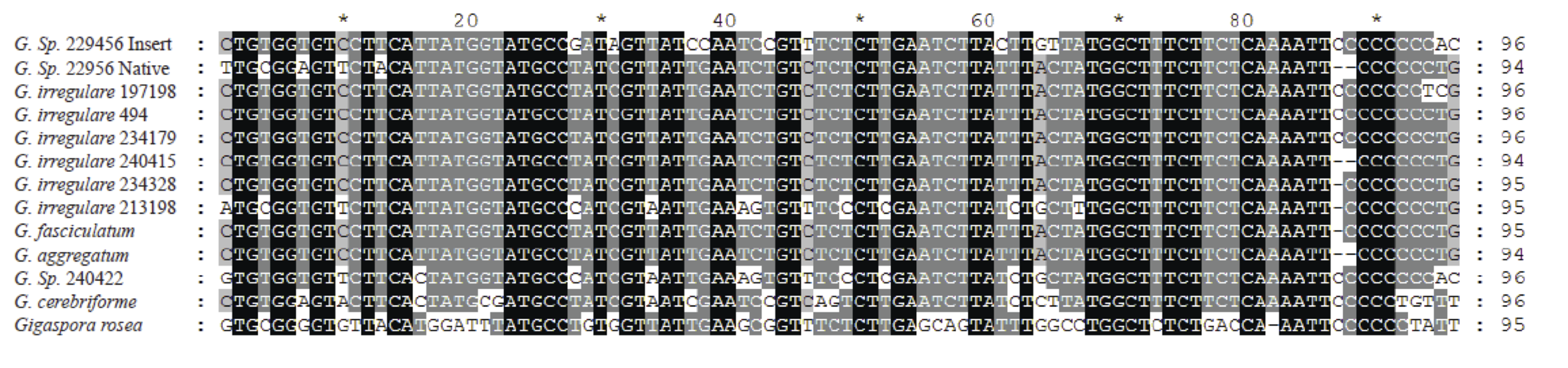

Supplement: Figure S3 — Multiple DNA sequence alignment of numerous AMF representatives of the cox2 native C-terminals along with the Glomus sp. 229456 putative foreign inserted C*-terminal. (TIF) [file pone.0060768.s003.tif]

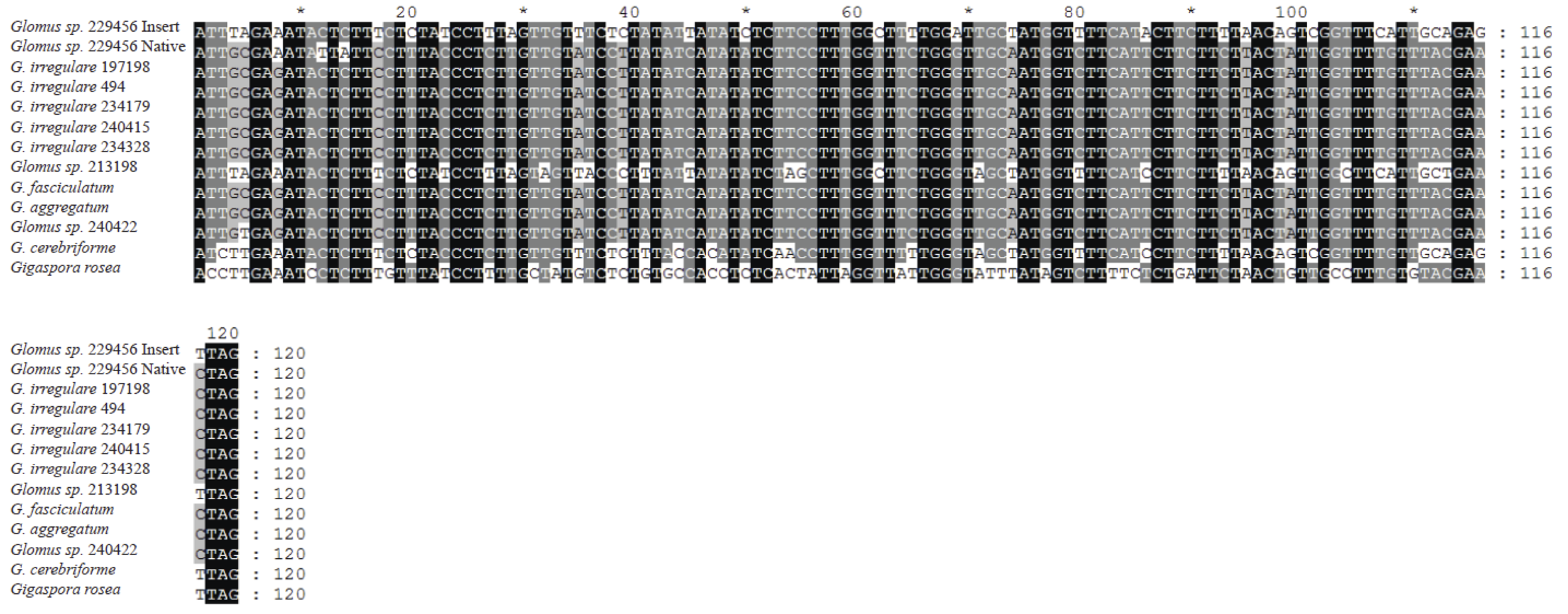

Supplement: Figure S4 — Multiple DNA sequence alignment of numerous AMF representatives of the nad3 native C-terminals along with the Glomus sp. 229456 putative foreign inserted C*-terminal. (TIF) [file pone.0060768.s004.tif]
