# Supplementary material for: Rapid Mitochondrial Genome Evolution through Invasion of Mobile Elements in Two Closely Related Species of Arbuscular Mycorrhizal Fungi
Source: PLoS One. 2013 Apr 18;8(4):e60768. doi: 10.1371/journal.pone.0060768 (PMC3630166; doi:10.1371/journal.pone.0060768)
Supplement: Table S1 — Sequence identity matrix of the atp6 native C-terminals along with the Glomus sp. 229456 putative foreign inserted C*-terminal. (DOC) [file pone.0060768.s005.doc]

**Table S1** Sequence identity matrix of the *atp6* native C-terminals along with the G*lomus sp. 229456* putative foreign inserted C*-terminal.

| **Seq->** | **Gsp229456**  **Insert** | **Gsp229456**  **Native** | **Gi197198** | **Gi494** | **Gi234179** | **Gi240415** | **Gi234328** | **Gsp213198** | **fascicula** | **aggregatum** | **Gsp240422** | **cerebri** | **G._rosea** |
| --- | --- | --- | --- | --- | --- | --- | --- | --- | --- | --- | --- | --- | --- |
| **Gsp229456**  **Insert** | ID | 63.5% | 68.1% | 68.1% | 68.1% | 68.1% | 68.1% | 74.1% | 68.1% | 68.1% | 68.1% | 56.1% | 50.3% |
| **Gsp229456**  **Native** | 63.5% | ID | 91.2% | 91.2% | 91.2% | 91.2% | 91.2% | 72.5% | 91.2% | 91.2% | 90.8% | 56.6% | 49.0% |
| **Gi197198** | 68.1% | 91.2% | ID | 100.0% | 100.0% | 100.0% | 100.0% | 71.7% | 100.0% | 100.0% | 99.6% | 56.0% | 48.5% |
| **Gi494** | 68.1% | 91.2% | 100.0% | ID | 100.0% | 100.0% | 100.0% | 71.7% | 100.0% | 100.0% | 99.6% | 56.0% | 48.5% |
| **Gi234179** | 68.1% | 91.2% | 100.0% | 100.0% | ID | 100.0% | 100.0% | 71.7% | 100.0% | 100.0% | 99.6% | 56.0% | 48.5% |
| **Gi240415** | 68.1% | 91.2% | 100.0% | 100.0% | 100.0% | ID | 100.0% | 71.7% | 100.0% | 100.0% | 99.6% | 56.0% | 48.5% |
| **Gi234328** | 68.1% | 91.2% | 100.0% | 100.0% | 100.0% | 100.0% | ID | 71.7% | 100.0% | 100.0% | 99.6% | 56.0% | 48.5% |
| **Gsp213198** | 74.1% | 72.5% | 71.7% | 71.7% | 71.7% | 71.7% | 71.7% | ID | 71.7% | 71.7% | 71.7% | 63.9% | 59.3% |
| **fascicula** | 68.1% | 91.2% | 100.0% | 100.0% | 100.0% | 100.0% | 100.0% | 71.7% | ID | 100.0% | 99.6% | 56.0% | 48.5% |
| **aggregatum** | 68.1% | 91.2% | 100.0% | 100.0% | 100.0% | 100.0% | 100.0% | 71.7% | 100.0% | ID | 99.6% | 56.0% | 48.5% |
| **Gsp240422** | 68.1% | 90.8% | 99.6% | 99.6% | 99.6% | 99.6% | 99.6% | 71.7% | 99.6% | 99.6% | ID | 56.0% | 48.5% |
| **cerebri** | 56.1% | 56.6% | 56.0% | 56.0% | 56.0% | 56.0% | 56.0% | 63.9% | 56.0% | 56.0% | 56.0% | ID | 63.7% |
| **G._rosea** | 50.3% | 49.0% | 48.5% | 48.5% | 48.5% | 48.5% | 48.5% | 59.3% | 48.5% | 48.5% | 48.5% | 63.7% | ID |
