# Supplementary material for: Rapid Mitochondrial Genome Evolution through Invasion of Mobile Elements in Two Closely Related Species of Arbuscular Mycorrhizal Fungi
Source: PLoS One. 2013 Apr 18;8(4):e60768. doi: 10.1371/journal.pone.0060768 (PMC3630166; doi:10.1371/journal.pone.0060768)
Supplement: Table S2 — Sequence identity matrix of the atp9 native C-terminals along with the Glomus sp. 229456 putative foreign inserted C*-terminal. (DOC) [file pone.0060768.s006.doc]

**Table S2** Sequence identity matrix of the *atp9* native C-terminals along with the *Glomus sp. 229456* putative foreign inserted C*-terminal.

| **Seq->** | **Gsp229456**  **Insert** | **Gsp229456**  **Native** | **Gi197198** | **Gi494** | **Gi234179** | **Gi240415** | **Gi234328** | **Gi213198** | **fascicula** | **aggregatum** | **Gsp240422** | **cerebri** | **G._rosea** |
| --- | --- | --- | --- | --- | --- | --- | --- | --- | --- | --- | --- | --- | --- |
| **Gsp229456**  **Insert** | ID | 70.5% | 72.5% | 72.5% | 72.5% | 72.5% | 72.5% | 80.3% | 72.5% | 72.5% | 74.5% | 74.5% | 70.5% |
| **Gsp229456**  **Native** | 70.5% | ID | 98.0% | 98.0% | 98.0% | 98.0% | 98.0% | 84.3% | 98.0% | 98.0% | 96.0% | 74.5% | 72.5% |
| **Gi197198** | 72.5% | 98.0% | ID | 100.0% | 100.0% | 100.0% | 100.0% | 86.2% | 100.0% | 100.0% | 98.0% | 76.4% | 70.5% |
| **Gi494** | 72.5% | 98.0% | 100.0% | ID | 100.0% | 100.0% | 100.0% | 86.2% | 100.0% | 100.0% | 98.0% | 76.4% | 70.5% |
| **Gi234179** | 72.5% | 98.0% | 100.0% | 100.0% | ID | 100.0% | 100.0% | 86.2% | 100.0% | 100.0% | 98.0% | 76.4% | 70.5% |
| **Gi240415** | 72.5% | 98.0% | 100.0% | 100.0% | 100.0% | ID | 100.0% | 86.2% | 100.0% | 100.0% | 98.0% | 76.4% | 70.5% |
| **Gi234328** | 72.5% | 98.0% | 100.0% | 100.0% | 100.0% | 100.0% | ID | 86.2% | 100.0% | 100.0% | 98.0% | 76.4% | 70.5% |
| **Gsp213198** | 80.3% | 84.3% | 86.2% | 86.2% | 86.2% | 86.2% | 86.2% | ID | 86.2% | 86.2% | 84.3% | 78.4% | 80.3% |
| **fascicula** | 72.5% | 98.0% | 100.0% | 100.0% | 100.0% | 100.0% | 100.0% | 86.2% | ID | 100.0% | 98.0% | 76.4% | 70.5% |
| **aggregatum** | 72.5% | 98.0% | 100.0% | 100.0% | 100.0% | 100.0% | 100.0% | 86.2% | 100.0% | ID | 98.0% | 76.4% | 70.5% |
| **Gsp240422** | 74.5% | 96.0% | 98.0% | 98.0% | 98.0% | 98.0% | 98.0% | 84.3% | 98.0% | 98.0% | ID | 78.4% | 72.5% |
| **cerebri** | 74.5% | 74.5% | 76.4% | 76.4% | 76.4% | 76.4% | 76.4% | 78.4% | 76.4% | 76.4% | 78.4% | ID | 68.6% |
| **G._rosea** | 70.5% | 72.5% | 70.5% | 70.5% | 70.5% | 70.5% | 70.5% | 80.3% | 70.5% | 70.5% | 72.5% | 68.6% | ID |
