# Supplementary material for: Rapid Mitochondrial Genome Evolution through Invasion of Mobile Elements in Two Closely Related Species of Arbuscular Mycorrhizal Fungi
Source: PLoS One. 2013 Apr 18;8(4):e60768. doi: 10.1371/journal.pone.0060768 (PMC3630166; doi:10.1371/journal.pone.0060768)
Supplement: Table S3 — Sequence identity matrix of the cox2 native C-terminals along with the Glomus sp. 229456 putative foreign inserted C*-terminal. (DOC) [file pone.0060768.s007.doc]

**Table S3** Sequence identity matrix of the *cox2* native C-terminals along with the *Glomus sp. 229456* putative foreign inserted C*-terminal.

| **Seq->** | **Gsp229456**  **Insert** | **Gsp229456**  **Native** | **Gi197198** | **Gi494** | **Gi234179** | **Gi240415** | **Gi234328** | **Gsp213198** | **fascicula** | **aggregatum** | **Gsp240422** | **cerebri** | **G._rosea** |
| --- | --- | --- | --- | --- | --- | --- | --- | --- | --- | --- | --- | --- | --- |
| **Gsp229456**  **Insert** | ID | 81.2% | 87.5% | 88.5% | 88.5% | 86.4% | 87.5% | 79.1% | 87.5% | 86.4% | 83.3% | 79.1% | 63.5% |
| **Gsp229456**  **Native** | 81.2% | ID | 90.6% | 92.7% | 92.7% | 94.6% | 93.6% | 85.2% | 93.6% | 94.6% | 81.2% | 78.1% | 66.6% |
| **Gi197198** | 87.5% | 90.6% | ID | 97.9% | 97.9% | 95.8% | 96.8% | 83.3% | 96.8% | 95.8% | 84.3% | 82.2% | 65.6% |
| **Gi494** | 88.5% | 92.7% | 97.9% | ID | 100.0% | 97.9% | 98.9% | 85.4% | 98.9% | 97.9% | 85.4% | 82.2% | 66.6% |
| **Gi234179** | 88.5% | 92.7% | 97.9% | 100.0% | ID | 97.9% | 98.9% | 85.4% | 98.9% | 97.9% | 85.4% | 82.2% | 66.6% |
| **Gi240415** | 86.4% | 94.6% | 95.8% | 97.9% | 97.9% | ID | 98.9% | 85.2% | 98.9% | 100.0% | 83.3% | 80.2% | 64.5% |
| **Gi234328** | 87.5% | 93.6% | 96.8% | 98.9% | 98.9% | 98.9% | ID | 86.3% | 100.0% | 98.9% | 84.3% | 81.2% | 65.6% |
| **Gsp213198** | 79.1% | 85.2% | 83.3% | 85.4% | 85.4% | 85.2% | 86.3% | ID | 86.3% | 85.2% | 92.7% | 73.9% | 62.5% |
| **fascicula** | 87.5% | 93.6% | 96.8% | 98.9% | 98.9% | 98.9% | 100.0% | 86.3% | ID | 98.9% | 84.3% | 81.2% | 65.6% |
| **aggregatum** | 86.4% | 94.6% | 95.8% | 97.9% | 97.9% | 100.0% | 98.9% | 85.2% | 98.9% | ID | 83.3% | 80.2% | 64.5% |
| **Gsp240422** | 83.3% | 81.2% | 84.3% | 85.4% | 85.4% | 83.3% | 84.3% | 92.7% | 84.3% | 83.3% | ID | 77.0% | 61.4% |
| **cerebri** | 79.1% | 78.1% | 82.2% | 82.2% | 82.2% | 80.2% | 81.2% | 73.9% | 81.2% | 80.2% | 77.0% | ID | 59.3% |
| **G._rosea** | 63.5% | 66.6% | 65.6% | 66.6% | 66.6% | 64.5% | 65.6% | 62.5% | 65.6% | 64.5% | 61.4% | 59.3% | ID |
