# Supplementary material for: Rapid Mitochondrial Genome Evolution through Invasion of Mobile Elements in Two Closely Related Species of Arbuscular Mycorrhizal Fungi
Source: PLoS One. 2013 Apr 18;8(4):e60768. doi: 10.1371/journal.pone.0060768 (PMC3630166; doi:10.1371/journal.pone.0060768)
Supplement: Table S4 — Sequence identity matrix of the nad3 native C-terminals along with the Glomus sp. 229456 putative foreign inserted C*-terminal. (DOC) [file pone.0060768.s008.doc]

**Table S4** Sequence identity matrix of the *nad3* native C-terminals along with the *Glomus sp. 229456* putative foreign inserted C*-terminal.

| **Seq->** | **Gsp229456**  **insert** | **Gsp229456**  **native** | **Gi197198** | **Gi494** | **Gi234179** | **Gi240415** | **Gi234328** | **Gsp213198** | **fascicula** | **aggregatum** | **Gsp240422** | **cerebri** | **G_rosea** |
| --- | --- | --- | --- | --- | --- | --- | --- | --- | --- | --- | --- | --- | --- |
| **Gsp229456**  **insert** | ID | 73% | 74% | 74% | 74% | 74% | 74% | 88% | 74% | 74% | 74% | 85% | 61% |
| **Gsp229456**  **native** | 73% | ID | 98% | 98% | 98% | 98% | 98% | 73% | 98% | 98% | 97% | 75% | 63% |
| **Gi197198** | 74% | 98% | ID | 100% | 100% | 100% | 100% | 73% | 100% | 100% | 99% | 76% | 63% |
| **Gi494** | 74% | 98% | 100% | ID | 100% | 100% | 100% | 73% | 100% | 100% | 99% | 76% | 63% |
| **Gi234179** | 74% | 98% | 100% | 100% | ID | 100% | 100% | 73% | 100% | 100% | 99% | 76% | 63% |
| **Gi240415** | 74% | 98% | 100% | 100% | 100% | ID | 100% | 73% | 100% | 100% | 99% | 76% | 63% |
| **Gi234328** | 74% | 98% | 100% | 100% | 100% | 100% | ID | 73% | 100% | 100% | 99% | 76% | 63% |
| **Gi213198** | 88% | 73% | 73% | 73% | 73% | 73% | 73% | ID | 73% | 73% | 73% | 83% | 61% |
| **fascicula** | 74% | 98% | 100% | 100% | 100% | 100% | 100% | 73% | ID | 100% | 99% | 76% | 63% |
| **aggregatum** | 74% | 98% | 100% | 100% | 100% | 100% | 100% | 73% | 100% | ID | 99% | 76% | 63% |
| **Gsp240422** | 74% | 97% | 99% | 99% | 99% | 99% | 99% | 73% | 99% | 99% | ID | 77% | 64% |
| **cerebri** | 85% | 75% | 76% | 76% | 76% | 76% | 76% | 83% | 76% | 76% | 77% | ID | 68% |
| **G_rosea** | 61% | 63% | 63% | 63% | 63% | 63% | 63% | 61% | 63% | 63% | 64% | 68% | ID |
